# Some Details of the Scheme

**Published:** 1914-07-04

**Authors:** 


### THE NEW ROYAL HOSPITAL FOR SICK CHILDREN, GLASGOW.

### Some Details of the Scheme.

Sir John Burnet and Dr. Mackintosh were left quite unhampered when the committee of the Royal Hospital for Sick Children, Glasgow, entrusted them with the responsible task of evolving a new hospital for the accommodation and treatment of 300 sick children. At the outset much trouble was taken in regard to the site, no less than eight different sites within the city boundary being examined before the present was finally selected. It contains high ground of ample area for the present buildings and for future extensions. It is situated in the western quarter of Glasgow, on the north bank of the Clyde near to its junction with the river Kelvin. The site slopes up from the east side, which is protected by existing buildings of a good class; it falls suddenly on the north, south, and west boundaries, where the immediate proximity of the two rivers mentioned prevents it from being hampered by future buildings. The site has the further advantage of being close to the main lines of railways and tramways, and is within easy walking distance of the University, the Western Infirmary, and their medical schools. The total cost of the site, buildings and equipment complete, it is estimated, will be about £140,000. Nearly the whole of this large sum has been obtained at an inclusive cost of less than half per cent., a remarkable instance of sound hospital finance aided by the whole-hearted co-operation of an intelligent and enlightened body of citizens.

The buildings now completed provide accommodation for 200 cots, which makes the inclusive maximum cost £700 per cot. It is stated, however, that when the buildings are extended to accommodate 300 children the cost per bed will be materially reduced. It is interesting to note that the work was commenced in 1911, so that three years have been occupied in completing the new buildings. The buildings are in blocks widely separated to ensure the maximum of sunshine and fresh air, and intercommunicate by corridors only two storeys in height. It will be seen from the plan that the new administration block is placed in the centre, with the admission and ward pavilions (which run north and south) on either side of the administration block and behind it. The central building is 150 feet long; the buildings are of brick, with white limestone facings. All the blocks, except the administration block, have flat roofs and can be used for exercise or sleeping accommodation, or to enable the children's cots to be there placed in the open air when the conditions of the climate are suitable. To facilitate these uses lifts have been provided and are large enough to take the cots from the wards to the roof, where shelters have also been placed. The largest wards contain eighteen cots each, being 90 feet in length and 27 feet wide. One pavilion contains wards of fourteen cots. Everything has been done by the designers to make the interior of the hospital as bright and attractive as possible. Some of the papers have claimed that this hospital is the largest children's hospital in the kingdom, but in fact the Hospital for Sick Children, Great Ormond Street, London, contains 240 patients' beds.

The site measures 14 acres or thereabouts, and the designers have placed grass plots between the pavilions and wherever it has been possible to fit them in. There can be no doubt that this newest children's hospital will create widespread interest amongst all who are familiar with the latest hospital equipment.

Two notable features of the plan are: (1) that each large ward is provided with its own isolation section, which consists of three rooms divided by glass partitions where children can be safely retained when infection is feared; (2) an admission block in a separate building which is equipped with means to enable a child to be thoroughly examined and cleansed before admission, and the clothing disinfected and returned to the friends in a condition to prevent the possibility of any danger of contagion to the remaining members of the family.

### Points on the Plans.

The plans of this hospital have a twofold and special interest. First, they are the joint work of Sir John Burnet, LL.D., and Dr. D. J. Mackintosh, M.V.O., of the Western Infirmary, Glasgow; and, secondly, these gentlemen have been round, we believe, to see not only the largest children's hospitals in the United Kingdom but the Continental ones also, so that it will be interesting for specialists to study the plans and inspect the buildings with a view to discovering if any and what novel features they present. Having regard to the importance of these special features we have invited the two gentlemen responsible for the conception and plans of this hospital to supply us with a statement embodying the points which they regard of importance and novelty, and the underlying principles which have governed them in its evolution.

Looking at the plan as a whole it will be observed that the outlook from the entrance portico and administration building windows is towards the boiler-house, laundry, and mortuary blocks. Site considerations may have influenced the choice of a position for these blocks, and we learn there is a great declivity at this end of the site; still it is not usual to put such blocks with the destructor in front of the main buildings in planning a new, up-to-date hospital.

The administration block consists of five storeys, of which four are devoted to the nurses' home. We propose to give a separate plan of the home on a future occasion. It is not usual, nor would it be desirable, in a large general hospital to have the board-room and office on the same floor and in the same building as the sisters' sitting and the nurses' drawing and writing rooms. In a children's hospital the meetings of the board may be less frequent and the business of the hospital can usually be transacted away from the hospital buildings, as we presume will be the case at Glasgow.

It is noticeable that, whereas the resident medical officers are to have on the ground floor two sitting and two bed rooms, and three sitting and three bed rooms on the first and second floors also, with ample bath and sanitary accommodation, the matron and the porter have each been provided with a house, according to the directions on the plan which has been issued by authority. Why are the sitting-rooms of the residents on the ground floor smaller than the bedrooms?

We observe that, whereas the sisters' rooms—is not this provision contrary to modern opinion and recent practice in up-to-date hospitals?—in Block C are well lighted and airy, the sisters' rooms in the larger blocks, D 1, 2, and 5, are imperfectly lighted, and must, we fancy, prove in Glasgow dark and unattractive during the majority of days in each year. We hope, therefore, that it may be possible to change this, because if the sister is to have a room at all adjacent to her ward it should be hygienically efficient, light, airy, and as attractive as possible.

We hope that due attention has been paid to the ventilation of the linen-rooms, which are usually defective on this point.

We can discover no sanitary blocks in connection with any of the ward pavilions. The only provision in the ward blocks appears to be a sink-room, with of course bathrooms. The sink-room contains one w.-c., and there is also a nurses' w.-c. adjacent to it. The sink-room is situated inside the block, and the closets and soil-pipes are inside too, but connected with pipe and vent shafts placed in a separate tower. This arrangement of the sanitary conveniences is a reversion to an old type of construction which we thought had been abandoned by general consent on hygienic grounds. Special merits are claimed for it by the designers.

We should like to know why the laundry block contains no provision for the reception, isolation, and disinfection of foul linen.

The mortuary block is attractive. It consists of three storeys. In the basement is the mortuary proper, with a workshop and refrigerator. On the ground floor the post-mortem, the chapel, and the museum are placed; and upstairs is a well-lighted and ample laboratory.

Finally, the operation block is a disappointment, having regard to the position of the anæsthetising-room in relation to the vestibule and theatres and the nurses' room. The plan generally would seem to be too crowded, and should both theatres be occupied at the same time the working of this block is likely to give rise to difficulties which should be avoided in planning so important a unit.

### Features of the General Plan.

The arrangement of the various pavilions and blocks on the plan as a whole—with the possible exception of the site chosen for the mortuary and laundry blocks, with the destructor already alluded to—is distinctly good. They permit of the freest circulation of air, of the completest isolation for any one and for all the blocks, and of the easy administration of the whole establishment from end to end. How far it is desirable to confine the verandah accommodation to the proportionately small area possible at the end of each ward pavilion is a matter which may have been governed in the present case by a knowledge of the atmospheric and weather conditions, which are factors that have to be severely borne in mind in the case of a great city like Glasgow and its surroundings.

We are glad to see that provision has been made for a dietetic kitchen, which should be of great value, providing it is placed in the responsible hands of a thoroughly capable and technically qualified sister. The same official might have charge of the milk-preparation rooms, which are another important feature of the kitchen block, and also of Ward Block 3.

The ward adjuncts are numerous and important. They include in the larger blocks sink, linen, and test rooms, in addition to a sisters' and a day room, a bathroom, a kitchen, a ward-maids' closet and two small wards, one with two beds. Ward Block C for nurslings has a surgical-dressings room and a nursing mothers' ward, but no small wards. A spacious conservatory connects the administration block with the kitchen or refectory block, and with its fountain this winter garden should be capable of being made attractive and helpful.

Taken as a whole we approve the plan and shall look forward with interest to an inspection of the buildings, which we hope to find in thorough working order. The gentlemen responsible for the plan are to be complimented upon the details and general results, which we have little doubt will be found to give complete satisfaction to the managers and staff. We congratulate Sir John Burnet and Dr. Mackintosh on the completion of a set of pavilions of such promise, and we propose to publish a full description of them after we have been through the hospital and seen it at work.

